# Population-level salt intake in the WHO European Region in 2022: a systematic review

**DOI:** 10.1017/S136898002200218X

**Published:** 2023-12

**Authors:** Edwin Jit Leung Kwong, Stephen Whiting, Anne Charlotte Bunge, Yana Leven, Joao Breda, Ivo Rakovac, Francesco Paolo Cappuccio, Kremlin Wickramasinghe

**Affiliations:** 1 World Health Organization European Office for the Prevention and Control of Noncommunicable Diseases, 9 Leontyevsky Pereulok, Moscow 125009, Russian Federation; 2 Melbourne School of Population and Global Health, University of Melbourne, Melbourne, VIC, Australia; 3 Stockholm Resilience Centre, Stockholm University, Stockholm, Sweden; 4 World Health Organization Regional Office for Europe, Copenhagen, Denmark; 5 University of Warwick, WHO Collaborating Centre for Nutrition, Warwick Medical School, Coventry, UK

**Keywords:** Salt, Na, Europe, Non-communicable diseases, Systematic review

## Abstract

**Objective:**

The WHO recommends that adults consume less than 5 g of salt per day to reduce the risk of CVD. This study aims to examine the average population daily salt intake in the fifty-three Member States of the WHO European Region.

**Design:**

A systematic review was conducted to examine the most up-to-date salt intake data for adults published between 2000 and 2022. Data were obtained from peer-reviewed and grey literature, WHO surveys and studies, as well as from national and global experts.

**Setting:**

The fifty-three Member States of the WHO European Region.

**Participants:**

People aged 12 years or more.

**Results:**

We identified fifty studies published between 2010 and 2021. Most countries in the WHO European Region (*n* 52, 98 %) reported salt intake above WHO recommended maximum levels. In almost all countries (*n* 52, 98 %), men consume more salt than women, ranging between 5·39 and 18·51 g for men and 4·27 and 16·14 g for women. Generally, Western and Northern European countries have the lowest average salt intake, whilst Eastern European and Central Asian countries have the highest average. Forty-two percentage of the fifty-three countries (*n* 22) measured salt intake using 24 h urinary collection, considered the gold standard method.

**Conclusions:**

This study found that salt intakes in the WHO European Region are significantly above WHO recommended levels. Most Member States of the Region have conducted some form of population salt intake. However, methodologies to estimate salt intake are highly disparate and underestimations are very likely.

High salt^1^ consumption is a major cause of hypertension (high blood pressure), which increases the risk of CVD such as stroke, myocardial infarction, heart and renal diseases^(1)^. Modelling suggests that 3 million deaths and 70 million disability-adjusted life years globally are attributed to high Na intake^(2)^. In the WHO European Region, CVD are the leading cause of death, premature mortality and disability^(3,4)^.

A dose–response relationship exists between the amount of salt consumed and both blood pressure and CVD^(5–7)^. Reductions in salt intake lead to reductions in systolic and diastolic blood pressure, in female and male adults, across ethnic groups and for both hypertensive and normotensive populations^(8–10)^. Therefore, population-level salt reduction is recommended as it is one of the most cost-effective measures for reducing the non-communicable diseases (NCD) burden and improving population health, by lowering the incidence of CVD, including strokes^(11)^. This is especially important during the COVID-19 pandemic, as modelling has suggested that one in five people worldwide have increased risk of severe COVID-19 if infected, due to underlying conditions such as CVD^(12)^.

Salt intake is also directly associated with BMI and the prevalence of overweight and obesity, which further amplifies the significance of promoting reduction in salt consumption as a means to address both the NCD and COVID-19 pandemics^(13)^. Therefore, NCD prevention, and more specifically population-wide salt reduction, should be a component of cities’ and countries’ COVID-19 recovery strategies, in order for societies to become healthier, more resilient and prepared for future pandemics^(14,15)^.

Salt reduction has been identified as a WHO Best Buy for the control and prevention of NCD in order to achieve Sustainable Development Goal target 3.4 to ‘reduce by one third premature mortality from non-communicable diseases’^(16)^. Therefore, the WHO recommends that adults consume no more than 5 g of salt (approximately 2 g of Na)/d to reduce the risk of CVD^(17)^. Furthermore, as a part of the WHO Global Action Plan to reduce the burden of NCD, in 2013 the World Health Assembly agreed to a global target of a 30 % reduction in population salt intake by 2025, through the implementation and scaling up of comprehensive salt reduction strategies^(18)^.

Previous estimates indicate that salt intake around the world is nearly twice the WHO recommended limit, and whilst Member States of the WHO European Region have demonstrated significant leadership and progress in efforts to reduce salt consumption, salt intake across the Region appears to be far above the recommended level^(19–24)^. A unified source of data on the population salt intake of every country in the Region can provide sound evidence to guide the development and monitoring of salt reduction policies. This study aims to examine the most up-to-date population salt intake data in the fifty-three Member States of the WHO European Region, providing a comprehensive overview of the existing data, highlighting gaps and issues in the evidence base and methodologies used.

## Methods

We conducted a systematic review following the preferred reporting items for systematic reviews and meta-analyses (PRISMA) reporting guidelines^(25)^; a protocol had not been published *a priori*, nor was this review registered.

### Search strategy

We conducted a systematic search in the electronic databases MEDLINE, Scopus and Web of Science in February 2022 for peer-reviewed articles published between 1 January 2000 and 31 January 2022. Search strings were devised to reflect population salt intake measurements and the respective countries of the WHO European Region. Two authors (EJLK and ACB) performed the abstract and full-text screening independently, using the open-access online tool CADIMA^(26)^, and resolved any contradictory assessments. Data were also acquired from surveys conducted by WHO, such as the questionnaire circulated amongst members of the European Salt Action Network (ESAN), as well as the NCD Country Capacity Survey, both conducted in 2019 by the WHO Europe NCD Office. Further data were obtained from studies conducted by WHO and from national surveys via programme leaders in national institutes and health ministries. When necessary, information was verified with national experts, and global experts in salt reduction reviewed all results. A detailed outline of the search strategy is provided in online supplementary material, Supplemental Appendix 1.

### Inclusion/exclusion criteria

As the main objective of this study was to identify the best available and up-to-date data on salt consumption in the fifty-three Member States of the WHO European Region, the studies were assessed against several criteria. Overall, we established the studies’ reliability and validity with the following criteria: method of measurement, sample population characteristics, year of the study and sample size. Twenty-four hour urinary collection is considered the gold standard in assessing population Na intake^(27)^. Other methods (biomarkers in spot urine collections, dietary surveys) are either flawed^(28–31)^ or grossly inaccurate^(32,33)^. Hence, we prioritised inclusion of studies that utilised 24 h urinary collection. However, in countries where 24 h urinary collection was not used, we selected studies using other methods.

We conducted a quality assessment of the measurement methods applied in the included studies, using the grading scale developed by Powles and colleagues^(34)^, with slight modifications to reflect the inclusion of spot urine collection as a method of salt intake measurement for this paper. There are nine levels to this grading scale, with nine being the highest possible score, reflecting the best evidence (detailed in online supplementary material, Supplemental Appendix 2). We used this quality assessment to prioritise inclusion of studies that did not use 24 h urinary collection, where studies with a higher score were selected. Where possible, studies which have a representative population sample, have larger sample sizes or are more recent in time are also prioritised.

We included only studies published since 2000 and limited inclusion only to countries in the WHO European Region (*n* 53). No language limits were imposed. In the case where there are multiple sources for one country, only one study deemed to be the most accurate and reliable estimation of the population average salt intake was included. We excluded studies that did not report salt or Na intake of an adult population^2^.

### Data extraction

The available data were collected from reports by EJLK and categorised under the following headings.

1. Country of study.
2. Mean salt intake in grams per day, with separate headings for men and women if sex-disaggregated data were reported.
3. Sample size, with separate headings for men and women if sex-disaggregated data were reported..
4. SD of mean salt intake.
5. Age range of participants.
6. Year of data collection.
7. Method of measurement.

If Na intake was reported, it was converted to salt intake with the conversion of 1 g Na = 2·542 g salt, which is the ratio of the molecular weight of sodium chloride (58·44) to the molecular weight of Na (22·99)^(35,36)^. If Na intake was reported in mmol/d, it was converted to mg/d with the conversion of 1 mmol Na = 23 mg Na, as the molar mass of Na is 22·99 g/mol^(36)^.

### Statistical analysis

Results are reported as means, and SD or 95 % CI. Weighted means and SD (based on sample sizes and CI) were calculated if not provided. Means for the total population, if not provided, were calculated by weighting the male and female means according to the national sex ratio in the closest year to the year of data collection, and SD for the total population were calculated by weighting the male and female SD equally. All calculations were performed using Microsoft Excel 365 version 16.52. If the sample size is small (*n* < 100), a *t*-distribution was used to calculate SD from the CI, instead of a standard normal distribution. Forest plots, stratified by methods of assessment of salt intake, were also produced for the total population, as well as for males and females separately. The figures in this study were produced in R version 4.0.3, QGIS version 3.16 and Microsoft Excel 365 version 16.52.

## Results

**Fig. 1:**
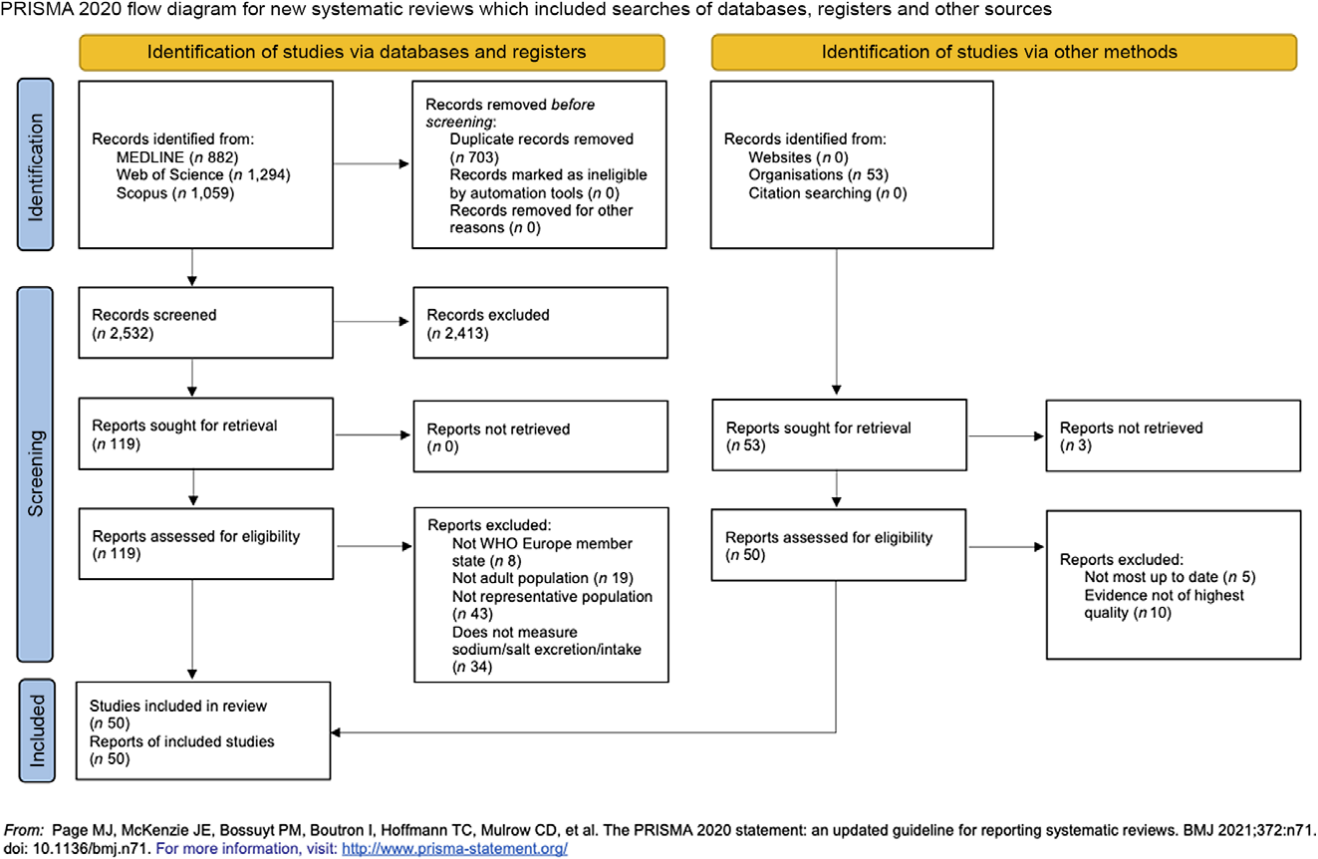
Preferred reporting items for systematic reviews and meta-analyses (PRISMA) flow diagram. Databases searched include MEDLINE, Scopus and Web of Science for peer-reviewed articles published between 1 January 2000 and 31 January 2022

We obtained data for the population mean daily salt intake for fifty of the fifty-three Member States of the WHO European Region. A total of 3235 records were retrieved from the initial search in peer-reviewed literature databases, of which 119 were assessed against the eligibility criteria at the full-text screening stage. This revealed fifteen eligible studies from peer-reviewed literature, which were then supplemented with grey literature (*n* 35), consisting of unpublished data obtained from national representatives and experts (*n* 8) and data from both WHO and national reports (*n* 27). The PRISMA flow diagram (Fig. 1) provides a detailed overview of the study and data selection conducted in this review. For the three Member States which did not have data available (Kyrgyzstan, Monaco, San Marino), they were imputed with proxy data from the closest neighbouring country with a similar ethnicity and cuisine.

### Study characteristics

The characteristics of the fifty studies identified are described in further detail in online supplementary material, Supplemental Appendix 2, with the year of data collection, method of measurement, sample size, age range of sample, sample population characteristics, sample representativeness and a quality assessment of each study’s measurement method included.

In summary, the fifty studies included in this paper were published between 2010 and 2021, and the dates of data collection were between 2007 and 2019. The characteristics of the sampled populations varied greatly, with considerable differences in sample sizes, ranging from a cohort of 113 from Ukraine to 6962 in Germany^(37,38)^. Notable differences were observed in sample characteristics, from cohorts of undergraduate students in Albania^(39)^ and Serbia^(40)^ to nationally representative samples in thirty studies. A higher proportion of women than men was also observed in most studies (*n* 40). The majority of studies included a wide age range of participants, with the exceptions of the undergraduate student cohorts, and the cohorts in the Belgian and Czech subsamples from the European Food Consumption Validation (EFCOVAL) project^(39,41–43)^.

### Mean salt intake across the WHO European Region

**Fig. 2:**
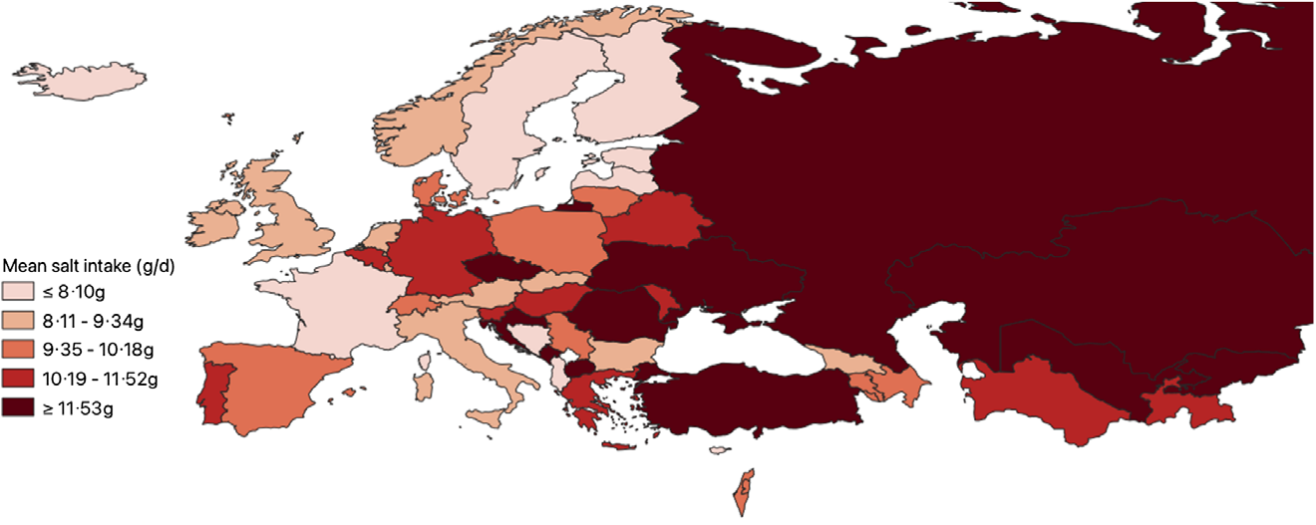
Map of mean population salt intake in the WHO European Region (2022). Displayed are the Member States of the WHO European Region divided into quintiles, based on their mean population salt intake. The detailed characteristics of the included studies can be found in online supplementary material, Supplemental Appendix 2

Data on mean salt intake were available in fifty out of fifty-three countries in the WHO European Region. Proxy data from neighbouring countries (Kazakhstan, France and Italy) were used to approximate three countries’ data (Kyrgyzstan, Monaco and San Marino), which did not have their own. Therefore, it was estimated that fifty-two out of fifty-three countries within the WHO European Region have a daily mean adult salt intake above the WHO recommended level of 5 g/d (Table 1). Salt intakes range between 5·39–18·51 g for men and 4·27–16·14 g for women. Forty-six countries have a population salt intake of at least 7·5 g/d, exceeding the recommended level by at least 50 %, and twenty-three countries have a population salt intake of at least 10 g/d, which is double the recommended level. Forty-seven out of fifty-three countries reported sex-specific data for mean adult salt intake. In almost every country, salt intake for men was higher than salt intake for women, with more than half of the countries (*n* 34) reporting at least a 2 g/d difference. Figure 2 shows the mean population salt intake of the Region stratified into quintiles, which are further detailed in online supplementary material, Supplemental Appendix 3.

**Table 1:** Daily mean salt intake in adults

Country	Total			Men			Women		
	Mean (g)	*n*	sd (g)	Mean (g)	*n*	sd (g)	Mean (g)	*n*	sd (g)
Albania^(39)^	6·05	289	3·01	–	37	–	–	252	–
Andorra^3^ ^(44)^	7·04	758	4·42	7·84	352	2·59	6·34	406	5·30
Armenia^(72)^	9·80	985	3·20	11·00	261	2·06	8·40	724	2·06
Austria^(73)^	8·32	615	6·43	8·98	231	5·63	7·69	384	7·14
Azerbaijan^(74)^	10·00	2487	3·82	11·40	1026	3·27	8·60	1461	2·93
Belarus^(75)^	10·60	4042	3·24	12·40	1687	3·14	9·00	2355	2·48
Belgium^(42)^	10·43	249	3·51	11·63	126	3·81	9·19	123	3·21
Bosnia and Herzegovina^(76)^	7·01	853	2·79	8·25	371	3·22	5·82	482	2·29
Bulgaria^4^ ^(77)^	8·13	306	3·66	8·98	153	3·63	7·31	153	3·68
Croatia^(78)^	11·75	504	4·41	13·37	–	4·34	10·37	–	4·04
Cyprus^5^	5·15	–	1·62	5·39	–	1·75	4·95	–	1·48
Czech Republic^(43)^	13·37	118	3·64	15·58	58	4·18	11·24	60	3·01
Denmark^(45)^	9·66	3016	3·25	11·19	1464	3·38	8·13	1552	2·39
Estonia^(79)^	5·21	2713	–	6·52	907	–	4·55	1806	–
Finland^(46)^	7·50	1655	–	8·70	780	–	6·40	875	–
France^(80)^	8·09	2121	3·42	9·15	887	3·25	7·09	1234	3·21
Georgia^(81)^	8·45	1252	2·28	9·65	352	2·13	7·35	900	1·80
Germany^(38)^	10·39	6962	0·20	11·20	3340	0·21	9·60	3622	0·18
Greece^(82)^	10·70	252	4·40	11·90	114	4·70	9·70	138	3·90
Hungary^6^	10·35	153	4·25	11·20	67	4·29	9·60	86	4·21
Iceland^(83)^	8·05	1312	3·46	9·59	632	3·81	6·61	680	2·30
Ireland^(84)^	9·30	488	4·10	10·40	306	4·30	7·40	182	2·70
Israel^(85)^	9·76	584	4·30	11·12	268	4·69	8·59	316	3·55
Italy^(86)^	8·47	1977	3·27	9·59	967	3·63	7·25	1010	2·87
Kazakhstan^(87)^	17·24	478	7·81	18·51	232	8·63	16·14	246	7·48
Kyrgyzstan^7^	17·24	N/A	7·81	18·51	N/A	8·63	16·14	N/A	7·48
Latvia^(47)^	7·34	1377	–	9·15	706	–	5·80	671	–
Lithuania^8^	10·00	888	5·30	11·70	466	5·80	8·40	422	4·10
Luxembourg^9^ ^(88)^	9·10	1326	4·08	10·51	617	4·48	7·88	709	3·24
Malta^10^	4·92	625	2·25	5·52	267	2·31	4·27	358	2·02
Moldova^(65)^	10·80	858	4·90	11·50	326	5·40	10·30	532	4·60
Monaco^11^	8·09	N/A	3·42	9·15	N/A	3·25	7·09	N/A	3·21
Montenegro^(66)^	11·60	639	5·60	13·90	285	6·30	9·90	354	4·30
Netherlands^(89)^	8·97	289	3·41	10·30	135	3·60	7·80	154	3·20
North Macedonia^(50)^	12·88	–	–	–	–	–	–	–	–
Norway^(90)^	8·98	493	3·48	10·40	241	4·08	7·57	252	2·76
Poland^12^	9·80	–	–	–	–	–	–	–	–
Portugal^(91)^	10·67	2568	3·78	10·85	1234	3·9	10·41	1344	3·64
Romania^(40)^	12·28	1970	3·90	–	936	–	–	1034	–
Russian Federation^(92)^	14·87	–	–	–	–	–	–	–	–
San Marino^13^	9·78	N/A	3·87	11·05	N/A	3·98	8·60	N/A	3·33
Serbia^(41)^	10·01	266	4·34	12·01	120	4·66	8·37	146	3·25
Slovakia^14^	8·50	–	–	–	–	–	–	–	–
Slovenia^(93)^	11·20	143	4·85	12·92	61	5·03	9·93	82	4·32
Spain^(94)^	9·82	418	4·60	11·48	196	4·78	8·36	222	3·88
Sweden^(48)^	7·93	1797	2·68	9·13	792	2·89	6·98	1005	2·06
Switzerland^(95)^	9·40	1350	3·80	10·80	663	4·20	8·00	687	3·30
Tajikistan^(96)^	10·40	2718	–	11·40	1100	–	8·80	1618	–
Turkey^(97)^	14·37	464	5·09	14·99	210	5·01	13·86	254	5·11
Turkmenistan^(98)^	10·30	2039	2·30	11·70	860	2·24	8·80	1179	1·75
Ukraine^(37)^	12·60	113	15·46	15·80	26	14·73	10·50	87	9·90
United Kingdom^(99)^	8·40	596	4·10	9·20	286	4·30	7·60	310	3·80
Uzbekistan^(100)^	14·88	598	3·49	14·86	252	3·52	14·90	346	3·51

### Estimation methods to measure mean population salt intake

**Fig. 3:**
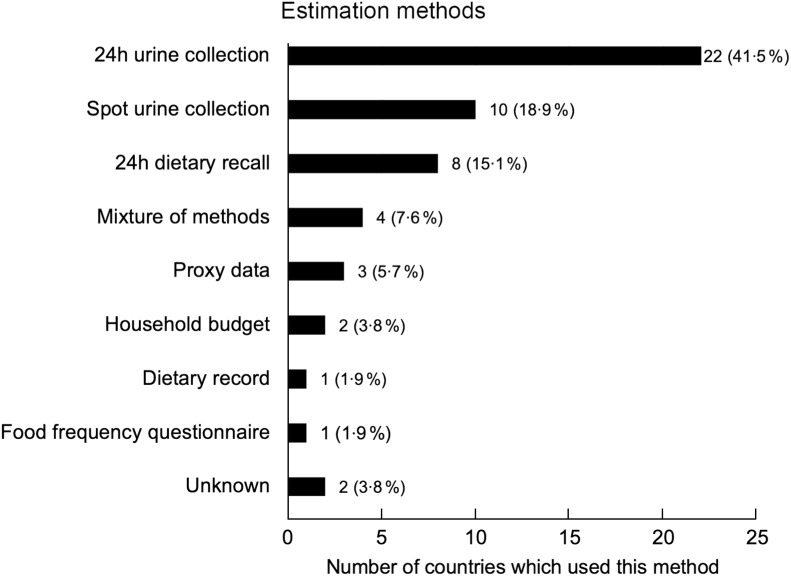
The different methods used to estimate salt intake data in the WHO European Region

Figure 3 shows the various methods of estimating salt intake data across the Member States of the WHO European Region. There is great variation, with nine different methods identified. Twenty-four hour urinary collection was the most common method (*n* 22), as we prioritised the inclusion of studies which used this gold standard method. Spot urine collection (*n* 10) and 24 h dietary recalls (*n* 8) were the next most common estimation methods. Andorra, Denmark, Finland and Latvia reported using a mixture of methods to estimate salt intake (*n* 4)^(44–47)^. Luxembourg was the only country to use only FFQ to estimate salt intake across the population (*n* 1), and only Sweden used dietary records to estimate salt intake (*n* 1)^(48,49)^. Household budget surveys were used to estimate salt intake in North Macedonia and Poland (*n* 2)^(50)^. We were unable to determine the estimation method for Cyprus and Slovakia (*n* 2), and primary data were not available for Kyrgyzstan, Monaco and San Marino (*n* 3).

**Fig. 4:**
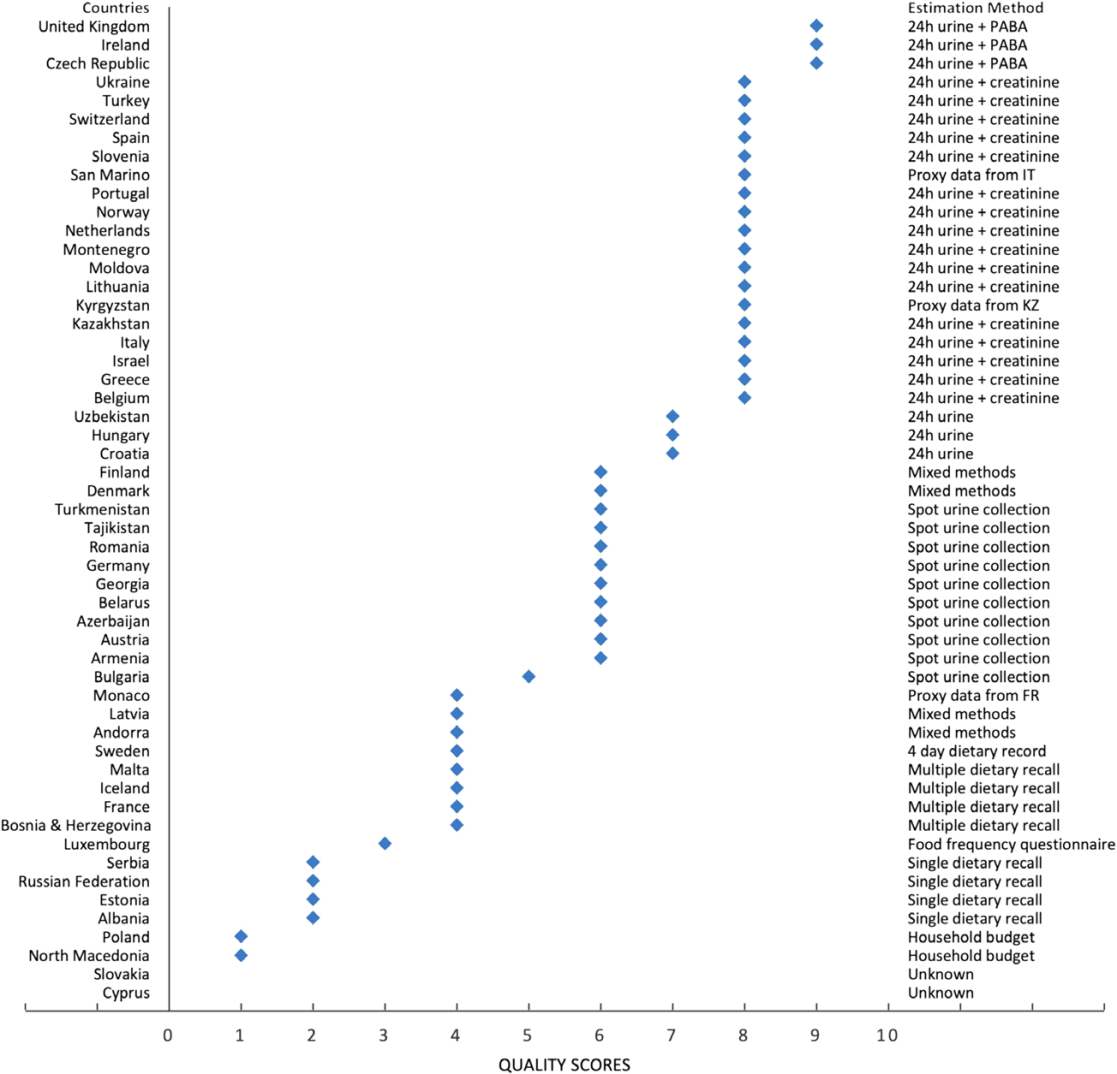
Quality of estimation method used for measuring salt intake for all studies included

In Fig. 4, we show the studies included, ranked by quality of assessment method, based on the collection method used. Further details of the studies and the quality assessments can be found in online supplementary material, Supplemental Appendix 2.

**Fig. 5:**
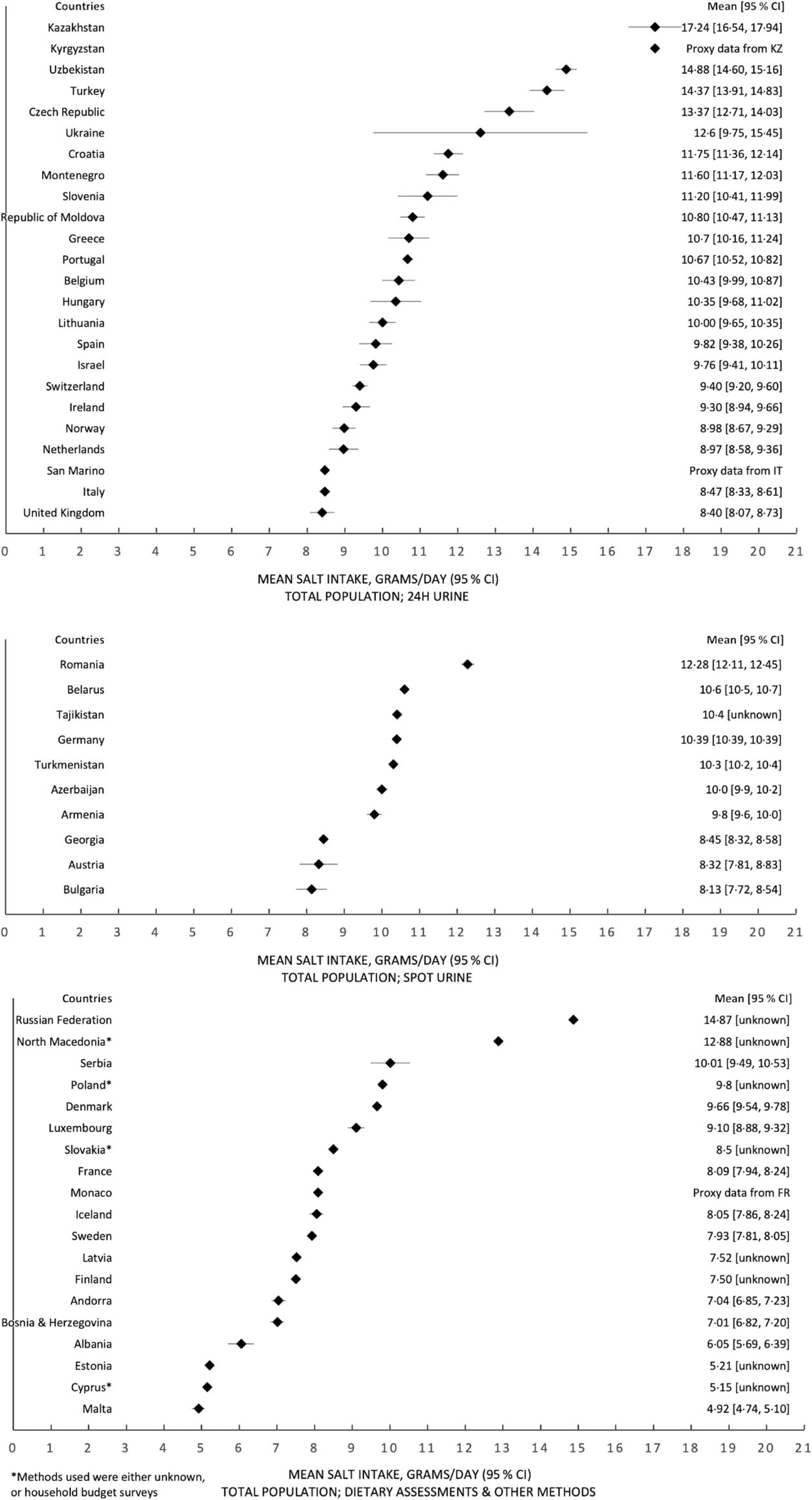
Forest plots, with estimates of the 95 % CI (except for a few countries which have provided these data), for the total population, split by estimation method of salt intake – 24 h urinary collection, spot urine collection and dietary assessments and all other methods

**Fig. 6:**
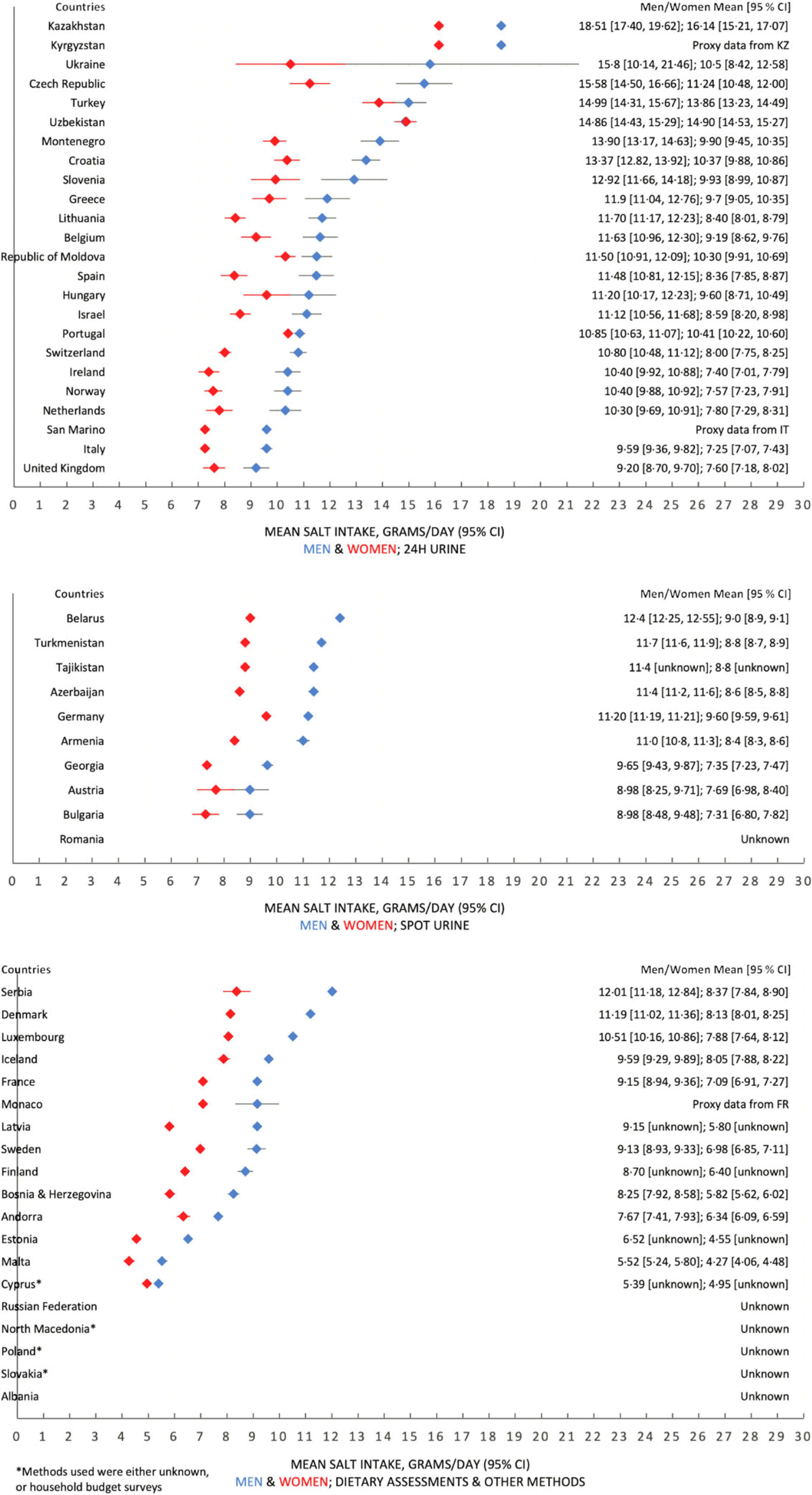
Forest plots, with estimates of the 95 % CI (except for a few countries which have provided these data), for male and female populations, split by estimation method of salt intake – 24 h urinary collection, spot urine collection and dietary assessments and all other methods

Figures 5 and 6 report forest plots of average salt consumption (and 95 % CI) by country and method of assessment. Whilst it is not possible to compare studies, there is a gradient, consistent in both men and women, in the direction of higher average estimates in countries using 24 h urinary collection compared with other methods. These trends would be expected from the evidence of regular underestimation of salt consumption by dietary methods (not allowing for discretionary sources of salt)^(32,33)^ and from a systematic bias when using spot urine collections, overestimating at lower level of salt consumption and underestimating at higher level^(12,51–55)^.

## Discussion

This review included fifty studies in the WHO European Region that reported Na or salt intakes amongst adult populations. Overall, we observed that the mean population salt intakes across the WHO European Region almost universally exceed the WHO recommended level of 5 g/d by significant margins. The level of salt intake across the fifty-three countries varied substantially, with a distinct East-West gradient in the Region, where salt intakes in Eastern European and Central Asian countries were higher than in Western and Northern European countries, highlighting the need for greater salt reduction efforts especially in those Member States. There were considerable differences observed between sexes, as men in thirty-four countries had a higher salt intake than women by over 2 g, and in eleven countries the difference was over 3 g. However, this sex difference is expected since salt intake is measured as total intake, not adjusted for energetic intake or body mass.

It is likely that the data in the lowest quintile (online supplementary material, Supplemental Appendix 3) are confounded by underestimations, as none of these studies (*n* 12) estimated salt intake with 24 h urinary collection, and dietary assessments in particular are likely to underestimate true salt intake^(32,33)^. For example, this is likely the case for Malta, which used dietary recalls to estimate salt intake. However, dietary recalls are unable to assess the amount of discretionary salt added to cooking and at the table. Therefore, it is highly possible that none of the fifty-three Member States of the WHO European Region meets the WHO recommended level of 5 g/d of mean population salt intake, as the one survey that reported figures below the recommended level is likely to have underestimated the true salt intake.

A broad evidence base was incorporated in this study by being flexible on the study types and methodologies included, as it was not limited to only published literature but also included grey literature and unpublished national and WHO reports. Therefore, we were able to demonstrate the state of population monitoring of salt intake across the WHO European Region, by both providing as complete a data set as possible and highlighting the many differing methodologies utilised across the fifty-three Member States.

Despite the fact that none of the Member States of WHO European Region is likely to have met the WHO recommended level of population salt intake, it is encouraging to see that fifty out of fifty-three countries were able to report its population salt intake in some form, which is an indication of the progress and success of scaling up measuring efforts, largely driven by the coordinated actions between WHO European Region, its Member States and the ESAN^(23)^. Only four countries had small sex-disaggregated sample sizes (*n* < 100), which is reassuring in terms of the included studies’ overall statistical precision and reliability. Finally, it is promising that the gold standard method of 24 h urinary collection was commonly used in the WHO European Region, with twenty-two out of fifty-three countries utilising this method to assess salt intake. Other methods not only tend to underestimate average population salt intake, but they will be less likely to detect small yet meaningful changes in average population salt consumption resulting from policy actions^(55–59)^, and risk deterring further action to achieve the WHO target of 5 g/d of salt intake^(58)^.

Whilst a key strength of this study is that a broad range of research with differing methodologies was incorporated, a significant limitation is that a majority of the included studies (*n* 28) utilised collection methods other than 24 h urinary collection, such as spot urine collection, dietary recall, food FFQ, dietary records, household budget surveys and also a mixture of some of these methods, which underestimate population-level salt intake^(32,60,61)^. For example, Freedman *et al.* found that 24 h dietary recalls underestimate Na intake by approximately 5–10 % and FFQ underestimate by approximately 30 %^(62)^. Hence, it is likely that the values reported from many of these studies not utilising 24 h urinary collection do not accurately reflect the actual salt intake of those countries’ populations. One study also used the Kawasaki formula to convert spot urine data to an estimate of 24 h urinary Na excretions^(40)^. However, this method has been proven to be inaccurate and systematically biased, with overestimations at lower levels of Na and underestimations at higher levels of Na, and a potential to skew the linear relationship demonstrated between levels of Na intake and mortality^(63,64)^. Additionally, according to the meta-analysis conducted by Lucko *et al*., approximately 93 % of dietary Na is excreted in urine, meaning that Na intake values estimated through 24 h urine collections should ideally be adjusted to most accurately reflect the true Na intake^(59)^. Furthermore, since there is no guarantee of the completeness of urine sample collections, validation markers such as creatinine ratio/total urinary creatinine can be useful in verifying the quality of the data collected, as can stringent standard operating procedures for the training of field workers and for quality control^(65–67)^.

Overall, of the fifty studies of adult salt intake identified in this article, only eighteen were given the highest measurement quality assessment scores of 8 or 9. This reveals that studies applying high-quality measurements of salt intake in WHO European Region are still lacking.

As no standardised method for estimating Na or salt intake was used between the studies included, the population salt intake values reported across the WHO European Region are not entirely immediately comparable. In order to counteract some of this inconsistency, we have ensured that where necessary, the same conversion factor to convert Na to salt was used across the studies included. In addition, not all data reported in the studies included were nationally representative, with 38 % of countries (*n* 20) either not providing information on the representativeness of their studies or did not conduct a nationally representative study. Some countries also had not conducted up-to-date monitoring of their salt intake, with some data being collected over 10 years ago, which suggests that some Member States in WHO European Region need to implement appropriate surveillance mechanisms more regularly for population salt intake.

Overall, the findings of this study indicate the need for Member States in the WHO European Region to conduct regular surveillance and monitoring of population salt intake to provide more up-to-date data. There is also still an overreliance on traditional nutritional surveys such as 24 h dietary recalls and FFQ to estimate salt intake. Further support, for example, by providing training on the use of WHO tools, such as the model protocol for measuring population salt intake using 24 h urinary data collections and implementation of STEPS surveys^(68)^, should be prioritised to support Member States’ surveillance and monitoring efforts. The usage of the gold standard method of 24 h urinary collection should be strongly encouraged in studies designed to accurately measure population-level salt intake and monitor changes over time.

It is hoped that policymakers in the WHO European Region and beyond can utilise the findings of this study to obtain information on salt consumption from comparable settings, as well as to determine the next steps in the monitoring of population salt consumption and the planning of future salt reduction strategies. Currently, the evidence demonstrates that reducing dietary salt reduces blood pressure in a linear relationship for both normotensive and hypertensive populations, as well as incidences of CVD^(1,5,7,9,10,69,70)^. This means that reducing population-level salt intake is an important step in decreasing mortality and improving population health. Therefore, this study, used in conjunction with the recent systematic review by Santos *et al.* on salt reduction efforts around the world, can provide useful information to help countries move towards the goal of a 30 % relative reduction in salt intake by 2025 by ensuring the scale up and more effective implementation of salt reduction initiatives^(18,71)^.

## Conclusion

Mean population salt intake in the WHO European Region is well above the WHO recommended level, with fifty-two out of fifty-three Member States exceeding it, and a strong likelihood of underestimation for the majority of studies included. However, it is encouraging to see that most Member States of the WHO European Region have conducted some form of population salt intake survey. To achieve the target level of salt intake recommended by the WHO, all Member States of the WHO European Region should scale up, or more effectively implement their salt reduction strategies, in accordance with the recommended interventions from the WHO Best Buys. In order to accomplish this, surveillance systems must be strengthened, and policy actions implemented across all sectors that can impact population salt intake. Future research, where practical, should utilise rigorous gold standard methods when collecting population salt intake data to ensure the highest degree of accuracy, comparability and validity.

## Footnotes

1 In this paper, salt is used to refer to different sources of Na salts (sodium chloride, sodium bicarbonate, monosodium glutamate, etc.), as Na is the chemical associated with risk. 5 g of sodium chloride (common salt) contains approximately 2 g (2000 mg) of Na.

2 There was considerable heterogeneity in both the definition of adult and the sampling age brackets across the studies included. For this study, persons 12 years and above were included, as three studies included populations below the age of 18 in a manner that made it challenging to disaggregate the data to only include persons 18 years and above.

3 Sample population includes persons 12 years and above.

4 Sample population includes persons 15 years and above.

5 Personal communication with Ms Eliza Markidou, Ministry of Health of the Republic of Cyprus. Unpublished study conducted in 2009 by the Cyprus Dietetic and Nutrition Association.

6 Personal communication with Dr Eszter Sarkadi Nagy, from the National Institute of Pharmacy and Nutrition, the successor of the National Institute for Food and Nutrition Science. Unpublished study conducted in 2010.

7 Proxy data used from Kazakhstan, due to lack of data in Kyrgyzstan.

8 Personal communication with Prof Marius Miglinas, Vilnius University. Unpublished study ‘NATRIJOD’, conducted between 2018 and 2020.

9 Supplemented by unpublished data, obtained from Dr Isabelle Ernens, Luxembourg Institute of Health.

10 Personal communication with Dr Daniel Cauchi, Ministry of Health, Malta. Unpublished study conducted between 2015 and 2017.

11 Proxy data used from France, due to lack of data in Monaco.

12 Calculations of the Department of Food and Nutrition Economics of Institute of Food and Nutrition, based on unpublished data from 2016, from the Department of Social Research and Living Conditions of the Central Statistical Office.

13 Proxy data used from Italy, due to lack of data in San Marino.

14 Personal communication with Dr Jozef Suvada, Ministry of Health, Slovak Republic.

## Supplementary material

For supplementary material accompanying this paper visit https://doi.org/10.1017/S136898002200218X
